# Antioxidant Vitamins and Their Use in Preventing Cardiovascular Disease

**DOI:** 10.3390/molecules15118098

**Published:** 2010-11-09

**Authors:** Dan Farbstein, Adena Kozak-Blickstein, Andrew P. Levy

**Affiliations:** Technion Faculty of Medicine, Technion Israel Institute of Technology, Haifa, Israel; E-Mails: alevy@tx.technion.ac.il (A.P.L.); kozy72@gmail.com (A.K.B.)

**Keywords:** vitamin E, antioxidants, vitamin C, atherosclerosis, CVD

## Abstract

Atherosclerosis remains one of the leading causes of death in Western populations. Subsequent to the discovery that oxidative stress plays a pivotal role in the development and progression of atherosclerosis, vitamins C and E, along with other antioxidants, were studied as potential therapies for the disease. However, while *in vitro* and *in vivo* studies showed promising antiatherogenic effects for vitamins C and E, clinical trials in which patients were given high doses of vitamin E or C showed no benefit and even possible harm. This review will attempt to summarize the known mechanistic data regarding the biochemical effects of vitamins C and E and their relevance to atherosclerosis, and offer an explanation for the failure of clinical trials to show that supplementation with these vitamins provides any benefit when given indiscriminately. We provide one example of how pharmacogenomics may be used to identify a sub-population which may indeed benefit from antioxidant supplementation.

## 1. Introduction

Atherosclerosis remains one of the leading causes of death in Western societies. Interventional therapies have focused on lowering the levels of low density lipoprotein (LDL) cholesterol which is closely correlated with the risk of atherosclerotic vascular disease. Experimental data suggesting that LDL oxidation may be an important process in the development of the atherosclerotic plaque led to the notion that decreasing oxidative stress may help prevent the disease or attenuate its progression [1]. Various antioxidants, and among them vitamins C and E, have attracted much attention regarding their ability to modulate the progression of atherosclerosis but clinical trials in which high dose supplements of these vitamins have been investigated have shown no benefits, and even possible harm with this intervention. However, *in vitro* and *in vivo* studies showing anti-atherogenic effects of these antioxidants on cells of the vessel wall raise questions regarding the ability of the trials conducted to reveal their true antiatherogenic potential. This review will give an update on the current knowledge regarding the atheroprotective effects of vitamins C and E, and will attempt to explain the failure of clinical trials to demonstrate benefit from vitamin supplementation. This will be done partially by demonstrating the unique pharmacogenetic relationship between vitamin E and the haptoglobin (Hp) phenotype in the setting of diabetes mellitus (DM).

## 2. Vitamin E

Vitamin E is a group of eight antioxidant lipophilic molecules, four of which are tocopherols and four of which are tocotrienols. It is mostly found in green vegetables, grains, nuts and various vegetable oils, as well as in eggs and milk. Although it is commonly known today for its antioxidant properties, the first biological role attributed to vitamin E was its necessity for fetal survival [2]. Today vitamin E is known to possess many biological properties, including antioxidant activity and the ability to modulate protein function and gene expression.

### 2.1. Structure and Localization

As mentioned, all vitamin E compounds are lipophilic. The lipophilicity of the compounds is attributed to their hydrophobic tail, a saturated phytyl chain in the tocopherols and an unsaturated phytyl chain in the tocotrienols. The antioxidant activity (see below) is attributed to the chromanol group, whose methylation differs among members of the vitamin E group. α-tocopherol, which is the most abundant vitamin E *in vivo*, is methylated on the 5^th^, 7^th^ and 8^th^ carbon of the chromanol ring [3]. Being a lipophilic molecule, vitamin E is most abundant in lipid phase compartments such as the plasma membrane and lipoproteins. It is also found in the membranes of cellular organelles and most notably in the lysosome and the Golgi membrane, where its concentration is more than ten times higher than in other membranes [4].

### 2.2. Absorption and Metabolism

Being a lipophilic molecule, vitamin E is absorbed in the gut via micelles, and then incorporated into chylomicrons [5]. When it reaches the circulation, vitamin E is transferred to other lipoproteins by the action of phospholipid transfer protein (PLTP) and to cells by the action of PLTP and lipoprotein lipase (LPL) [6]. Vitamin E is also taken up and re-distributed by the liver, with the uptake of chylomicrons and the release of very low density lipoproteins (VLDL) [7]. The liver can also secrete vitamin E by the α-tocopherol-transfer protein (α-TTP), which is highly specific for α-tocopherol [8] and mediates its transfer to various lipoproteins [9]. Vitamin E levels are tightly regulated by enzymatic activity of the CYP enzymes, the activity of which changes in response to changes in plasma α-tocopherol levels. Other forms of vitamin E, such as γ-tocopherol, are also metabolized and excreted, but unlike α-tocopherol, they do not have a profound effect on CYP activity. α-Tocopherol can also be excreted in the bile via the Multi Drug Resistance (MDR) family of transporters [10].

### 2.3. Biological Functions of Vitamin E in Relation to Atherosclerosis

#### 2.3.1. Regulation of Cell Survival, Proliferation and Apoptosis

An inhibitory effect on protein kinase C (PKC) was one of the first established non-antioxidant functions of vitamin E to be identified [11]. Vitamin E was found to activate phospho-serine/threonine phosphatase 2A (PP2A), which is responsible for the dephosphorylation of PKC, a process that occurs on the cell membrane [12]. The most prominent effect of vitamin E mediated by PKC inhibition is the reduction of cell proliferation. This has been shown to occur in various cells [13], the inhibition of vascular smooth muscle cells (VSMCs) being most relevant to the attenuation of the atherosclerotic process [14,15]. Another signaling pathway which is subjected to modulation by vitamin E is the mitogen-activated protein kinase (MAPK) pathway. In VSMCs stimulated by oxidized LDL, vitamin E was shown to decrease MAPK activity and enhance cell survival [16]. Vitamin E was also shown to inhibit Protein Kinase B (PKB) and activate protein tyrosine phosphatase, both altering cell proliferation and survival [13].

#### 2.3.2. Enhancement of Endothelial Function

Vitamin E was shown to enhance various functions of the endothelium, including nitric oxide (NO) release, anti-thrombotic properties and vasodilation. As opposed to the inhibitory effect on arachidonic acid (AA) release and metabolism in other cells, most notably in macrophages, vitamin E leads to an increase in AA release by phospholipase A_2_ (PLA_2_) in endothelial cells. Although this effect is accompanied by a decrease in cyclo-oxygenase (COX) 1 and 2 activity, the net effect is an increase in the production of vasodilating prostanoids PGE_2_ and PGI_2_ [17]. Additionally, vitamin E was shown to enhance the phosphorylation of endothelial nitric oxide synthase (eNOS) on serine 1177, resulting in an amplification of its action [18,19]. These results translate to increased levels of NO metabolites following vitamin E treatment [20]. However, the effect of vitamin E treatment on endothelial function is still unclear, as some studies have found an improvement in endothelial function following vitamin E treatment [21,22,23,24], but others have not [25,26,27,28].

#### 2.3.3. Regulation of Inflammatory Processes

Vitamin E has been shown to inhibit several inflammatory processes which are known to take place during atherogenesis. Vitamin E inhibits the cellular adhesion process and decreases the expression of various adhesion molecules and chemokines by endothelial cells and leukocytes, both *in vitro,* in a response to a variety of noxious stimuli [29,30,31,32,33], and *in vivo* [34]. Humans receiving high dose vitamin E supplementation demonstrate a decrease in soluble adhesion molecules [20,35,36]. Additionally, vitamin E was shown to suppress the secretion of pro-inflammatory cytokines such as tumor necrosis factor-α (TNF-α) [37] and interleukin-1 β (IL-1β) [38,39]. Scavenger receptors, such as CD36, known to be important for oxidized LDL uptake by macrophages, are down regulated by vitamin E [40,41]. Finally, vitamin E inhibits the activity of inducible NOS (iNOS) and NADPH oxidase, thereby inhibiting the macrophage respiratory burst [42,43].

#### 2.3.4. Antioxidant Function

Vitamin E is classified as an antioxidant due to its ability to scavenge lipid radicals and terminate oxidative chain reactions. It can terminate radical chain reactions by interacting with the lipid peroxyl radical, preventing it from generating a new radical and perpetuating the chain reaction by oxidizing other lipids. This is due to the rate constant of the reaction between lipids and lipid peroxyl radicals, which is 1,000-fold lower than the rate constant of the reaction between α-tocopherol and lipid peroxyl radicals (10^2^ M^-1^S^-1^ compared to 10^5^–10^6^ M^-1^S^-1^). It is unlikely that vitamin E would interfere with the radical chain reaction in other stages. The radical chain reaction is usually initiated by water soluble molecules, where vitamin E is sparse due to its lipophilic nature. Its interaction with lipid radicals is unlikely since the rate constant of the reaction between lipid radicals and oxygen is 100-1,000-fold higher compared to that of lipid radicals and vitamin E. Following its oxidation, vitamin E can be recycled back to its native unoxidized form by various soluble antioxidants such as vitamin C and ubiquinol. This process prevents the accumulation of vitamin E radicals and their subsequent peroxidation of lipids [44], and is considered by some to be critical for the antioxidant activity of vitamin E [45]. It has been suggested that all of the other biological functions of vitamin E are actually a result of its antioxidant activity [46].

### 2.4. Vitamin E in Clinical Studies

While *in vitro* and *in vivo* studies demonstrate a wide variety of anti-atherogenic effects for vitamin E, these were not translated into the clinical setting as large trials showed no beneficial effect for vitamin E supplementation [47,48,49]. 

Overall, supplementation of vitamin E has been shown to cause an increase in mortality in a large meta-analysis [50]. One reason for the failure to show a beneficial effect for vitamin E supplementation may be the fact that it was indiscriminately given to a large population. However, as is true for any pharmaceutical agent, vitamin E supplementation would be predicted to show benefit only in those individuals in which it is needed. Demonstrating this important concept of proper patient selection, when vitamin E was selectively given to DM patients with the Hp 2-2 phenotype, it appeared to provide a significant positive effect on CVD and overall mortality [51]. Additionally, a re-analysis of large clinical trials according to Hp phenotype has shown similar results [52,53]. This data is summarized in Table 1. The mechanisms underlying this pharmacogenetic effect between the Hp genotype and vitamin E were recently summarized in a comprehensive review [54].

## 3. Vitamin C

As opposed to vitamin E, vitamin C (L-ascorbate) is a hydrophilic molecule, and, therefore, it is found mostly in bodily fluids. Vitamin C is abundant in fruits and vegetables and they serve as the main source for dietary vitamin C intake. However, modern food processing methods lead to the loss vitamin C, as well as many other vitamins and nutrients [55]. Isolated in 1928, vitamin C was recognized as the bioactive molecule that was missing in the diet of sailors, causing scurvy [56]. Vitamin C is known to take part in many physiological processes, and has been proposed to have a beneficial or therapeutic role in immune responses, cardiovascular disease and cancer [55].

### 3.1. Chemistry of L-Ascorbate and Antioxidant Activity

L-Ascorbate’s unique structure that includes two adjacent hydroxyl groups and a carbonyl makes this molecule an excellent hydrogen or electron donor. Therefore, it takes part as a co-factor in many enzymatic reactions, and also acts as a plasma localized anti-oxidant. Once oxidized, ascorbate is turned into ascorbate free radical (AFR), a molecule that is relatively stable due to electron delocalization. Although AFR can donate another electron, it does not undergo further oxidation. Rather, it is reduced back to ascorbate via NADH-dependent and independent mechanisms. AFR accumulation, resulting from increased oxidative conditions, leads to a reaction between two AFR molecules that form one molecule of ascorbate and one molecule of dehydroascorbate (DHA). DHA itself can either be reduced back to ascorbate, or hydrolyzed to gulonic acid [57]. L-Ascorbate fulfills the requirements of an antioxidant, since it can react with radicals and terminate their reaction. Indeed, in the cellular environment where its concentrations are high and recycling mechanisms are abundant, L-ascorbate protects the cell from oxidative stress [58]. However, L-ascorbate radical can also serve as an electron donor, and actually accelerate redox reactions in the presence of transition metals such as iron or copper. Thus, in the atherosclerotic plaque where ferric iron is present, vitamin C might serve as a pro-oxidant rather than as an anti-oxidant [59].

### 3.2. L-Ascorbate Absorption, Reabsorption and Cellular Uptake

In most mammals, L-ascorbate is synthesized endogenously by the enzyme L-gulono-γ-lactone oxidase. However, in primates and guinea pigs a functional gene for this enzyme is absent and therefore, the only source of L-ascorbate in guinea pigs and primates is from the diet [57]. Absorption, reabsorption and cellular uptake of L-ascorbate are mediated by the sodium dependent vitamin C transporters (SVCTs). There are two families of these transporters, SVCT1 and SVCT2. Both of these transporters couple the entry of Na^+^ with that of L-ascorbate into cells, against its electrochemical gradient. Different tissues in the body express different types of SVCTs [60]: while both SVCT1 and SVCT2 are expressed in the gastrointestinal tract and mediate absorption of L-ascorbate [60], only SVCT1 is expressed in the kidney and mediates reabsorption. Thus, knockout of SVCT1 led to renal loss of L-ascorbate in mice and significantly decreased levels of L-ascorbate in plasma [57]. Endothelial cells express SVCT2 alone [61]. The SVCT2 transporter seems to be critical for normal development of blood vessels, as SVCT2^-/-^ mice present with petechia and ecchymoses in the brain shortly after birth [62]. The SVCTs are able to form a gradient of L-ascorbate of up to 1:50 [57]. This means that when plasma concentrations are as low as 30-60μM, cellular concentration of L-ascorbate can be as high as 4mM, concentrations that are needed for optimal production of Type IV collagen [63].

### 3.3. Effects of L-Ascorbate on Cells in the Vessel Wall and its Atheroprotective Properties

The atheroprotective properties of L-ascorbate arise not only from its ability to act as an antioxidant and to reduce vitamin E, but also from its effects on different cells of the vessel wall. 

#### 3.3.1. Induction of Endothelial Cell Proliferation:

L-Ascorbate has been shown to promote endothelial cell proliferation, and decrease growth inhibition and apoptosis induced by TNF-α, oxidative stress [64] and oxidized LDL [65]. The proliferative effect of L-ascorbate is thought to be mediated by its effect on Type IV collagen synthesis, which is an integral constituent of the basement membrane and is also responsible for endothelial adhesion. This was proven by showing a decrease in L-ascorbate’s proliferative action in the presence of *cis*-hydroxyprolyl (CHP), which inhibits the enzyme prolyl hydroxylase that is essential for the production of Type IV collagen in endothelial cells. Looking at intracellular signaling, L-ascorbate was shown to decrease p53 levels and increase phosphorylation of the cell cycle regulator Rb, thus rendering it inactive and enabling proliferation [64]. 

#### 3.3.2. Prevention of Endothelial Cell Apoptosis:

The ability of L-ascorbate to decrease apoptosis in oxidative and inflammatory conditions has been shown to be the result of inhibition of cytochrome C release from mitochondria and prevention of the activation of caspase 9. The same anti-apoptotic effect is exerted by NO as well, and indeed, inhibition of NO production by adding L-NMMA to the culture medium abolished the anti-apoptotic effects of L-ascorbate [66]. Furthermore, other studies have shown that L-ascorbate can potentiate the production of NO by stabilizing tetrahydrobiopterin, the co-factor required for the enzymatic reaction carried out by NOS. This has an effect not only on endothelial survival but also on the entire vessel wall, reducing oxidative stress and inflammation [67]. 

#### 3.3.3. Enhancement of Endothelial Function

Considering its net proliferative effect on the endothelium and its ability to enhance NO production, L-ascorbate is expected to reverse endothelial cell dysfunction. Indeed, several independent trials have shown that L-ascorbate effectively reverses endothelial cell dysfunction caused by hypercholesterolemia [68], hypertension, diabetes and atherosclerosis [57]. Reversal of endothelial cell dysfunction was not only shown after acute single intravenous dose of L-ascorbate, but also after chronic (one month) oral intake of the vitamin [69].

#### 3.3.4. Inhibition of Smooth Muscle Cell Proliferation

The atheroprotective effects of L-ascorbate are not restricted to the endothelium alone. Aside from the antiatherogenic effect exerted by the overlying endothelium, the smooth muscle cells of the vessel wall are directly affected by L-ascorbate. Most notably, L-ascorbate was shown to decrease smooth muscle cell proliferation in response to mildly oxidized LDL [65]. Furthermore, L-ascorbate was shown to induce smooth muscle cell differentiation *in vitro*. *In vivo* studies on rabbits that underwent balloon induced carotid injury showed that not only did L-ascorbate induce differentiation of smooth-muscle cells in the neointimal layer of the plaque, but it also prevented dedifferentiation of smooth muscle cells of the media [70]. This effect may have a crucial impact on plaque progression, as dedifferentiated smooth muscle cells cannot only proliferate, but also differentiate to macrophages and thus intensify the inflammation in the vessel wall and accelerate plaque growth. The prevention of neointimal growth was shown in a clinical trial in which oral intake of L-ascorbate resulted in larger luminal diameter and decreased need for another intervention four months after angioplasty [71].

### 3.4. Ability of L-ascorbate to Reduce Cardiovascular Events and Overall Mortality

While the studies presented suggest that L-ascorbate has an atheroprotective effect, clinical trials regarding its ability to reduce cardiovascular risk and overall mortality have not shown any benefit; L-ascorbate had no added effect in decreasing thickness of the carotid artery wall [72], nor was it able to attenuate coronary atherosclerotic progression [73]. In an eight-year trial that studied the ability of L-ascorbate and vitamin E (either each one alone, or both together) to prevent cardiovascular events, cerebrovascular events and overall mortality of healthy men or men suffering from cardiovascular disease, L-ascorbate did not show any benefit on any of the study endpoints [74].

## 4. Conclusion—Future Perspectives Regarding Antioxidant Supplementation

The studies presented here have emphasized the marked disparity between the anti-atherogenic effect of the antioxidant vitamins C and E shown in preclinical studies, and their inability to show beneficial effects in clinical trials. *In vitro* and *animal* studies may perhaps not accurately represent the biological processes in the human body. However, other explanations may exist for the discrepancy, pertaining to the methodology of the clinical trials. We have presented one example, involving the Hp genotype and vitamin E, wherein only a subset of patients may actually benefit from antioxidant vitamin E therapy. 

An additional problem with trials regarding antioxidant supplementation, is that the timing of their administration may be critical. Vitamin C has been shown to have beneficial effects on processes that occur in the early stages of atherosclerosis; it may prevent lesion formation in the first place or initial plaque growth by improving endothelial function and preventing the formation of the neointima. However, once the atherosclerotic plaque is already formed, the contribution of vitamin C to antiatherogenic processes may be negligible [57]. Most clinical trials were conducted on patients that already suffered from vascular disease [72,73]. Even among patients without a history of prior symptomatic CVD, the participants’ age in most of these studies was over 50, an age in which atherosclerotic lesions and plaques are certainly already present [74]. Trials enrolling younger participants are needed to examine whether vitamin C has an effect in the early stages of atherosclerosis. 

The dose and mixture of antioxidants that are given may also be critical. In the initial observational dietary studies that demonstrated strong apparent benefit from antioxidant vitamins, vitamins were obtained from fruits and vegetables. Naturally occurring antioxidant vitamins differ in their formulation (*i.e.,* synthetic vitamin E contains a mixture of stereoisomers while natural vitamin E contains only one stereoisomer) and in the relative concentrations of related molecules. The difference between the many different forms of vitamin E which occur in natural food substances and those that were used in failed clinical trials is striking.

Why might antioxidant supplementation be harmful for some populations? Oxidative processes are vital for normal cellular function, and have a pivotal role in various physiological systems, including normal vascular physiology. When given in pharmacological doses, which are much higher than doses that can be attained by dietary intake, antioxidants may attenuate both deleterious and beneficial oxidative processes. This may be the reason why clinical trials that use pharmacological doses of antioxidants do not show a beneficial effect on disease progression when given indiscriminately to all individuals regardless of their baseline level of oxidative stress.

## Figures and Tables

**Table 1:** Pharmacogenetic interaction between the Hp 2-2 genotype and vitamin E therapy.

Study	Outcome	Reference
***ICARE***	50% reduction in CVD following vitamin E treatment compared to placebo in Hp 2-2 diabetics.	[51]
***HOPE***	55% reduction in CV Death, 41% reduction in MI following vitamin E treatment compared to placebo in Hp 2-2 diabetics.	[52]
***WHS***	15% reduction in CVD following vitamin E treatment compared to placebo in Hp 2-2 diabetics.	[53]

ICARE: Israel Cardiovascular Events Reduction with vitamin E study; CVD: Cardiovascular Disease; Hp: Haptoglobin; HOPE: Heart Outcomes Prevention Evaluation MI: Myocardial Infarction; WHS: Women’s Health Study.
